# Assessment of factors affecting flicker ERGs recorded with RETeval from data obtained from health checkup screening

**DOI:** 10.1371/journal.pone.0284686

**Published:** 2023-04-24

**Authors:** Taiga Inooka, Taro Kominami, Shunsuke Yasuda, Yoshito Koyanagi, Junya Ota, Satoshi Okado, Ryo Tomita, Yasuki Ito, Takeshi Iwase, Hiroko Terasaki, Koji M. Nishiguchi, Shinji Ueno

**Affiliations:** 1 Department of Ophthalmology, Nagoya University Graduate School of Medicine, Nagoya, Japan; 2 Department of Ophthalmology, Kyushu University Graduate School of Medical Sciences, Fukuoka, Japan; 3 Department of Ophthalmology, Fujita Health University School of Medicine, Toyoake, Japan; 4 Department of Ophthalmology, Akita University Graduate School of Medicine, Akita, Japan; 5 Department of Ophthalmology, Hirosaki University Graduate School of Medicine, Hirosaki, Japan; Roskamp Institute, UNITED STATES

## Abstract

**Purpose:**

To determine the factors significantly associated with the amplitudes and implicit times of the flicker electroretinograms (ERGs) recorded with the RETeval system by analyzing the comprehensive data obtained during a health checkup screening.

**Methods:**

Flicker ERGs were recorded with the RETeval system from 373 individuals who had a normal fundus and optical coherence tomography images. The sex, age, anthropometric, ophthalmologic, and hematologic data were collected from all participants who were 40- to 89-years-of-age. Univariable and multivariable linear mixed effects regression analyses were performed to identify factors that were significantly associated with the implicit times and amplitudes of the RETeval flicker ERGs.

**Results:**

Univariable linear mixed effects regression analysis showed significant correlations between the implicit times and the best-corrected visual acuity, the age, the axial length, the blood sugar level, and the blood urea nitrogen level. Analyses by multivariable linear mixed effects regression identified that the axial length (β = 0.28), the age (β = 0.24), and the blood sugar level (β = 0.092) were three independent factors that were significantly correlated with the implicit times of the RETeval flicker ERGs. Univariable linear mixed effects regression analysis also showed significant correlations between the amplitudes of the RETeval flicker ERGs and the age, the platelet count, and the creatinine level. Multivariable linear mixed effects regression models identified the age (β = -0.092), the platelet count (β = 0.099), and the creatinine level (β = -0.12) as three independent factors that were significantly correlated with the amplitudes of the RETeval flicker ERGs. However, the smoking habits, body mass index, and the blood pressure were not significantly correlated with either the implicit times or amplitudes of the RETeval flicker ERGs.

**Conclusions:**

Our results indicate that the age and some ophthalmologic and hematologic findings but not the anthropometric findings were significantly associated with the implicit times and amplitudes of the RETeval flicker ERGs. Thus, clinicians should remember these factors when analyzing the RETeval flicker ERGs.

## Introduction

Full-field electroretinography (ERG) is an essential clinical test that provides an objective, quantitative measure of the retinal function in patients with various retinal diseases. Several protocols for the different testing procedures for recording ERGs have been recommended by the International Society of Clinical Electrophysiology of Vision (ISCEV) [1]. Among them is the protocol for recording the full-field flicker ERGs that are evoked by 30 Hz (acceptable range 28–33 Hz) stimuli for the assessment of the photopic pathway of the retina. Flicker ERGs represent the response of the cone pathway because rod photoreceptors cannot respond to stimulus frequencies above about 15 Hz [2]. Thus, analyses of the flicker ERGs are a relatively simple method to assess the photopic pathway. The recordings can be completed within 10–20 seconds, and it does not require a dark-adaptation period.

A relatively new ERG recording system called the RETeval system (LKC Technologies, Inc., Gaithersburg, MD, USA) has been introduced, and it has drawn the attention of clinicians because of its ease of use. This system is equipped with a small handheld ganzfeld dome for eliciting the ERGs, and the ERGs are picked-up by a single-use skin strip electrode array. This strip contains the active, the reference, and ground electrodes. The RETeval system can record ERGs without mydriasis because the RETeval system delivers stimuli with constant retinal luminance (photopic Td-s) by adjusting the luminance (photopic cd-s/m^2^) to compensate for changes in the pupillary area (mm^2^) in real time. These conditions make the recording of the flicker ERGs easier and more convenient for the patients and clinicians. Therefore, flicker ERGs recorded with the RETeval system have been used not only to evaluate retinal diseases, e.g., central retinal vein occlusion (CRVO) [3, 4], blue-cone monochromatism (BCM) [5], and retinopathy of prematurity (ROP) [6], but also for the screening of retinal diseases, e.g., for diabetic retinopathy [7–11].

To use RETeval as a screening tool, it is necessary to determine the factors affecting the amplitudes and implicit times of the RETeval flicker ERGs in normal eyes. The results of earlier studies have shown the influence of the axial length and pupillary area on the different components of the RETeval flicker ERGs [12, 13]. However, the correlations of the RETeval flicker ERGs with the hematologic tests and anthropometric data of normal individuals have not been determined.

Thus, the purpose of this study was to determine the significance of the correlations between the implicit times and the amplitudes of the RETeval flicker ERGs and the values of the hematologic tests and the anthropometric data of the participants. To accomplish this, we recorded RETeval ERGs from a large number of individuals undergoing a health checkup screening. We identified the factors significantly associated with the amplitudes and implicit times of the RETeval flicker ERGs.

## Materials and methods

### Study design

This was a prospective, single center study. All of the procedures conformed to the tenets of the World Medical Association’s Declaration of Helsinki and were approved by the Nagoya University Hospital Ethics Review Board. The subjects were volunteers who attended a basic health checkup screening that was supported by the local government of Yakumo town in 2015. All participants signed a written informed consent form after they were provided with information on the procedures to be used. This Yakumo Study was conducted in the town of Yakumo located in a rural area of southern Hokkaido, Japan. All subjects were ≥40-years-of-age and had undergone assessments of not only the ophthalmic parameters but also anthropometric assessments and hematologic tests.

### Protocols for general examinations related to current study

The fasting blood samples were collected through venipuncture and centrifuged within an hour of the collection. The serums were stored at -80°C until the assays were performed. Routine biochemical analyses were performed in the laboratory of the Yakumo Town Hospital. Anthropometric measurements of the body height and weight were obtained during the screening and used to calculate the body mass index (BMI, kg/m^2^). Ultrasound examinations were performed to measure the intima-media thickness (IMT) and to determine plaque formation in the carotid arteries as described in detail [14–17]. The mean value of the right and left max IMT was used for the statistical analyses.

### Protocols for ocular examinations

The best-corrected visual acuity (BCVA) was measured by an automatic vision tester (Nidek, NV-350, Gamagori, Aichi, Japan) with correcting eyeglasses based on the refractive error measured by the auto Ref/Keratometer (Nidek, TONOREF III, Gamagori, Aichi, Japan). Anterior segment examinations were performed by slit-lamp biomicroscopy by ophthalmologists. Color fundus photographs were taken with a nonmydriatic fundus camera (Canon, Canon CR-2, Tokyo, Japan), and the macular morphology was determined by OCT recordings (Nidek, RS-3000, Gamagori, Aichi, Japan).

All of the data were evaluated by ophthalmologists. The axial length was measured by partial coherence interferometry (Carl Zeiss Meditec, Inc. IOLMaster, Dublin, CA, USA). The intraocular pressure (IOP) was measured by noncontact tonometry (Nidek, TONOREF III, Gamagori, Japan).

**Fig 1:**
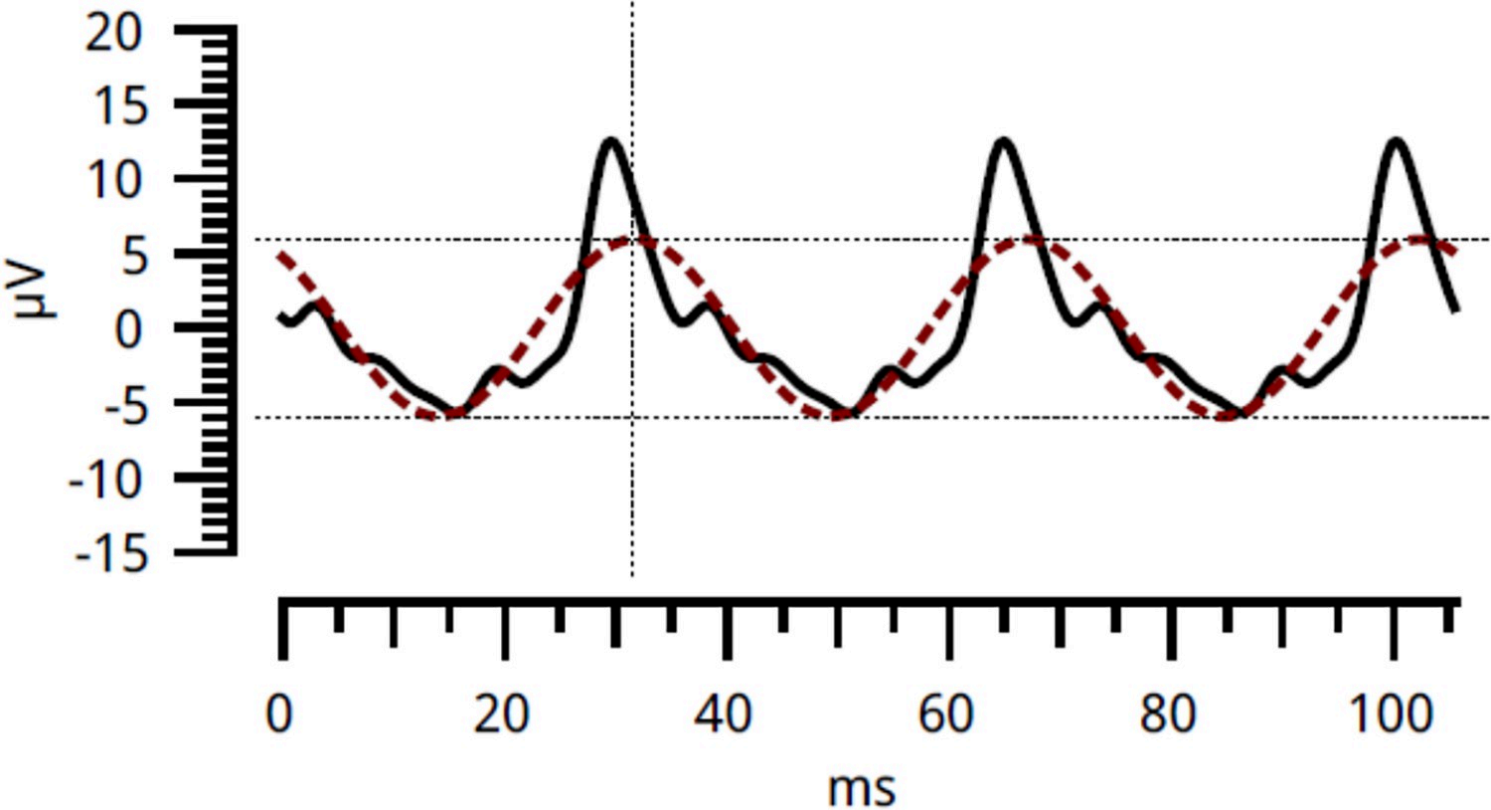
Examples of the flicker ERG recorded by the RETeval system. Two flicker ERGs of the fundamental component (colored dotted line) and the reconstructed flicker ERG waveform using the first eight harmonics (solid black line) are presented by this system.

The flicker ERGs were recorded with the RETeval system (LKC Technologies, Gaithersburg, MD, USA) without mydriasis and were elicited by white stimuli of 8 Td-second/m^2^. No background illumination was used. The frequency of the flicker stimulus was 28.306 Hz. The diameter of the pupil was measured automatically in real-time by the RETeval system and the diameter at which the equilibrium was reached was adopted. The fundamental components of the flicker ERGs were automatically measured and displayed by the RETeval system using a special algorithm based on discrete Fourier transformation and cross-correlation analysis (see Fig 1 for details) [18].

### Subjects

A total of 497 individuals took part in the ocular examinations. Of these, reliable flicker ERGs were recorded from 443 right eyes and 446 left eyes with the RETeval system. Among these, 110 right eyes and 114 left eyes were excluded because of known ocular diseases or abnormal findings in the color fundus photographs or OCT images. The reasons for the exclusion were, glaucoma or glaucoma suspect in 46 right eyes and 55 left eyes, drusen in 20 right eyes and 17 left eyes, epiretinal membrane in 11 right eyes and 10 left eyes, age-related macular degeneration in 11 right eyes and 8 left eyes, and other factors in 26 right eyes and 35 left eyes. In addition, there were some individuals who had multiple disease findings (see S1 Table for details) who were excluded. In the end, the findings in 333 right eyes and 332 left eyes from 373 individuals were used for the statistical analyses.

### Statistical analyses

We analyzed the data of the general condition including the sex, age, height, weight, systolic blood pressure (sBP), diastolic blood pressure (dBP), average blood pressure, Brinkman index, and hematologic test of 23 factors. We analyzed the data of the ophthalmic examinations including the BCVA, IOP, axial length, radius of the pupillary diameter, and the implicit times and amplitudes of the flicker ERGs (see S2 Table for details).

For the BCVA, the decimal values were converted to the logarithm of the minimum angle of resolution (logMAR) units for the statistical analyses. The body mass index (BMI) was calculated from the weight and height using the equation:

$$\mathrm{BMI}=\frac{\mathrm{weight}\;(\mathrm{kg})}{{\mathrm{height}\;(m)}^{2}}=10000\times \frac{\mathrm{weight}\;(\mathrm{kg})}{{\mathrm{height}\;(\mathrm{cm})}^{2}}$$

The average BP was calculated from the systolic and diastolic blood pressures using the equation: $\mathrm{average}\;\mathrm{BP}\;(\mathrm{mmHg})=\mathrm{Diastolic}\;\mathrm{BP}\;(\mathrm{mmHg})+\frac{\left(\mathrm{Systolic}\;\mathrm{BP}\;(\mathrm{mmHg})‐\mathrm{Diastolic}\;\mathrm{BP}\;(\mathrm{mmHg})\right)}{3}$

To assess the effects of the smoking habits, we calculated the Brinkman index as follows:

Brinkmanindex=Numberofcigarettessmoked/day×Yearsofsmoking

The pupillary area was calculated from the radius of the pupillary diameter using the equation:

$$\mathrm{pupillary}\;\mathrm{area}\;({\mathrm{mm}}^{2})=\pi \times {\left(\mathrm{radius}\;\mathrm{of}\;\mathrm{the}\;\mathrm{pupillary}\;\mathrm{diameter}\;(\mathrm{mm})\right)}^{2}$$

Because of the high correlation of the response variables between right and left eyes, linear mixed effects regression analyses with a random intercept were used to determine the factors which were significantly associated with the implicit times and amplitudes of the fundamental component of the flicker ERGs. The implicit times and amplitudes of the fundamental component of the flicker ERGs were used as the response variables, and the explanatory variables included the age, sex, height, BMI, systolic and diastolic BPs, average BP, Brinkman index, IOP, axial length, BCVA (log MAR units), pupillary area, and items of the hematologic test (up to 37 items).

Univariable models were first used and the items with *p-* values <0.05 after Holm-Bonferroni correction in the univariable models were further analyzed. The regression coefficient (β) and 95% confidence intervals (95% CI) were also calculated.

For further analyses, we built a multivariable model with backward stepwise selection using Akaike information criterion (AIC) for estimating the best predictive model in the selection of the explanatory variables. Standardized partial regression coefficient (β) were calculated for the independent variables after unifying the units of the variables by standardizing all variables to a mean 0 and variance 1. Unstandardized partial regression coefficient (B) and 95% confidence intervals (95% CI) were also calculated for presentation of the results of the final model with backward stepwise selection. The variance inflation factor (VIF) was also calculated to estimate the severity of the multicollinearity. In the analyses of the multivariable models, the results were considered statistically significant when the *p-* values were <0.05.

These analyses were performed with scikit-learn = 0.24.0 (https://scikit-learn.org/stable/install.html) based on python = 3.6.7 (https://www.python.org/downloads/release/python-367/) and lme4 = 1.1–30 (https://cran.r-project.org/web/packages/lme4/index.html) based on R = 4.4.2 (https://www.r-project.org/).

## Results

The main demographic information of the 665 eyes of 373 subjects is shown in Table 1. The mean age ± standard deviations [range] of the individuals analyzed was 62.5 ± 9.5 [40–89] years. The mean implicit time ± standard deviations [range] of the individuals analyzed was 32.0 ± 1.5 [27.3–37.5] ms and the mean amplitude ± standard deviations [range] was 9.73 ± 3.2 [1.8–20.4] μV. The results of the 23 hematologic tests are shown in S3 Table.

**Table 1:** Demographic information.

Number of eyes	665
Age (years)	62.5 ± 9.5 [40–89]
Sex (Male/Female)	286 / 379
BCVA (logMAR units)	0.050 ± 0.19 [-0.18–1.0]
IOP (mmHg)	13.5 ± 2.5 [6.3–20.7]
Axial length (mm)	23.8 ± 1.2 [20.72–28.08]
Pupillary area during ERG (mm^2^)	5.60 ± 2.38 [1.59–31.8]
Implicit times of flicker ERGs (msec)	32.0 ± 1.5 [27.2–37.5]
Amplitudes of flicker ERGs (μV)	9.73 ± 3.16 [1.8–20.4]
Height (cm)	157.9 ± 8.4 [136.0–181.9]
BMI (kg/m^2^)	23.5 ± 3.4 [15.7–40.9]
sBP (mmHg)	129.7 ± 20.6 [82–190]
dBP (mmHg)	75.6 ± 13.8 [43–133]
average BP (mmHg)	93.6 ± 15.3 [56.0–149.7]
Brinkman index (cigarettes・years/day)	282 ± 404 [0–2400]
IMT (mm)	0.97 ± 0.56 [0.4–3.1]
BS (mg/dL)	89 ± 14 [60–160]
HDL (mg/dL)	59.5 ± 14.5 [32–125]
BUN (mg/dL)	14.7 ± 3.8 [5.4–29.2]
Plt (10^4^/μL)	21.9 ± 4.9 [9.3–41.7]
Cre (mg/dL)	0.75 ± 0.17 [0.43–1.55]

BCVA, best-corrected visual acuity; IOP, Intraocular pressure; BMI, Body mass index; sBP, Systolic blood pressure; dBP, Diastolic blood pressure; IMT, Intima Media Thickness; BS, Blood sugar; HDL, High-density lipoprotein; BUN, Blood urea nitrogen; Plt, Platelet; Cre, Creatinine.

Data are expressed as the means ± standard deviations [range].

### Correlations between implicit times of flicker ERGs and other health checkup factors

First, we determined the significance of the correlations between the implicit times of the flicker ERGs and the 37 health checkup measures including the ophthalmic, anthropometric, and hematologic findings. The results of the univariable linear mixed effects model regression analyses with a random intercept are shown in Table 2. The factors that were significantly correlated with the implicit times of the RETeval flicker ERGs with *p-* values <0.05 after the Holm-Bonferroni method were the BCVA (logMAR units), axial length, the age, blood sugar level, and blood urea nitrogen (BUN) values.

**Table 2:** Univariable linear mixed effects model regression analysis between implicit times and explanatory variables.

	regression coefficient (β)	95% CI [0.025, 0.975]	*p-* value	*p-* value corrected using the Holm-Bonferroni method
BCVA (logMAR units)	1.2	0.59, 1.8	<0.001*	0.0044*
IOP (mmHg)	5.5×10^−2^	8.2×10^−3^, 0.10	0.022*	n.s.
Axial length (mm)	0.25	0.15, 0.35	<0.001*	<0.001*
Pupil Area (mm^2^)	1.5×10^−2^	-3.4×10^−2^, 6.4×10^−2^	0.55	n.s.
Sex-Male	0.19	-4.2×10^−2^, 0.43	0.11	n.s.
Sex-Female	-0.19	-0.43, 4.2×10^−2^	0.11	n.s.
Age (years)	3.9×10^−2^	2.7×10^−2^, 5.1×10^−2^	<0.001*	<0.001*
Height (cm)	7.6×10^−3^	-6.3×10^−3^, 2.1×10^−2^	0.28	n.s.
BMI (kg/m^2^)	4.7×10^−2^	1.2×10^−2^, 8.1×10^−2^	0.008*	n.s.
Systolic BP (mmHg)	4.3×10^−3^	-1.4×10^−3^, 9.9×10^−3^	0.14	n.s.
Diastolic BP (mmHg)	5.6×10^−4^	-7.9×10^−3^, 9.0×10^−3^	0.90	n.s.
average BP (mmHg)	2.9×10^−3^	-4.8×10^−3^, 1.1×10^−2^	0.46	n.s.
Brinkman index (cigarettes・years/​day)	-2.2×10^−5^	-3.1×10^−4^, 2.7×10^−4^	0.88	n.s.
IMT (mm)	0.24	2.9×10^−2^,0.45	0.026*	n.s.
WBC (10^3^/μL)	5.2×10^−2^	-2.7×10^−2^, 0.13	0.19	n.s.
RBC (10^4^/μL)	5.9×10^−4^	-2.3×10^−3^, 3.5×10^−3^	0.70	n.s.
Hb (g/dL)	2.8×10^−3^	-9.1×10^−2^, 9.7×10^−2^	0.95	n.s.
Ht (%)	-4.6×10^−4^	-3.2×10^−2^, 3.1×10^−2^	0.98	n.s.
MCV (fL)	1.0×10^−2^	-3.4×10^−2^, 1.4×10^−2^	0.39	n.s.
Plt (10^4^/μL)	-3.2×10^−3^	-2.7×10^−2^, 2.1×10^−2^	0.79	n.s.
HbA1c (%)	0.28	5.4×10^−2^, 0.51	0.016*	n.s.
BS (mg/dL)	1.4×10^−2^	5.3×10^−4^, 2.2×10^−2^	0.0015*	0.0495*
Protein (g/dL)	-0.14	-0.44, 0.16	0.36	n.s.
Alb (g/dL)	-0.43	-0.90, 5.4×10^−2^	0.083	n.s.
ALP (U/L)	8.7×10^−4^	-9.9×10^−4^, 2.7×10^−3^	0.36	n.s.
GOT (U/L)	9.5×10^−3^	-4.7×10^−3^, 2.4×10^−2^	0.19	n.s.
GPT (U/L)	4.7×10^−3^	-2.9×10^−3^, 1.2×10^−2^	0.22	n.s.
γGTP (U/L)	2.9×10^−3^	5.5×10^−4^, 5.2×10^−3^	0.016*	n.s.
Total cholesterol (mg/dL)	-1.9×10^−3^	-5.2×10^−3^, 1.5×10^−3^	0.28	n.s.
TG (mg/dL)	7.1×10^−4^	-1.2×10^−3^, 2.6×10^−3^	0.47	n.s.
HDL (mg/dL)	-7.4×10^−4^	-1.5×10^−2^, 6.6×10^−4^	0.073	n.s.
LDL (mg/dL)	-2.9×10^−3^	-6.7×10^−3^, 8.3×10^−4^	0.13	n.s.
BUN (mg/dL)	5.9×10^−2^	2.9×10^−2^, 9.0×10^−2^	<0.001*	0.0052*
Cre (mg/dL)	0.82	0.12, 1.5	0.023*	n.s.
Uric acid (mg/dL)	0.13	4.6×10^−2^, 0.22	0.0030*	n.s.
Ca (mg/dL)	-9.2×10^−2^	-0.51, 0.33	0.67	n.s.
CRP (mg/dL)	0.38	-0.16, 0.92	0.16	n.s.

CI, confidence interval; BCVA, best-corrected visual acuity; IOP, Intraocular pressure; BMI, Body mass index; BP, Blood pressure; IMT, Intima Media Thickness; WBC, white blood cell; RBC, red blood cell; Hb, hemoglobin; Ht, hematocrit; MCV, mean corpuscular volume; Plt, Platelet; HbA1c, Hemoglobin A1c; BS, Blood sugar; Alb, albumin; ALP, alkaline phosphatase; GOT, glutamic oxaloacetic transaminase; GPT, glutamic pyruvic transaminase; γGTP, γ- glutamyl transpeptidase; TG, triglyceride; HDL, high density lipoprotein cholesterol; LDL, low density lipoprotein cholesterol; BUN, Blood urea nitrogen; Cre, Creatinine; Ca, calcium; CRP, C-reactive protein; n.s., not significant.

* means *p*- value <0.05

Adjusted P values for factors that were not significant in univariable models are not shown because they are also not significant after Holm-Bonferroni adjustment.

These explanatory variables were then tested by multivariable linear mixed effects model regression analyses. Backward stepwise model selection using Akaike information criterion (AIC) showed that the linear mixed effects regression models with explanatory variables of the axial length, the age, and the blood sugar level were the best explainers for the variations of the implicit times: the axial length (*p* <0.001), the age (*p* <0.001), and blood sugar (*p* = 0.022) were significantly correlated with the implicit times of the fundamental components of the RETeval flicker ERGs (Table 3).

**Table 3:** Multivariable linear mixed effects model regression analysis between implicit times and explanatory variables.

	Standardized partial regression coefficient (β)	Partial regression coefficient (B)	95% CI [0.025, 0.975]	*p*- value	VIF
Axial length (mm)	0.28	0.39	0.29, 0.48	<0.001*	1.10
Age (years)	0.24	5.1×10^−2^	3.9×10^−2^, 6.3×10^−2^	<0.001*	1.14
BS (mg/dL)	9.2×10^−2^	9.5×10^−3^	1.3×10^−3^, 1.8×10^−2^	0.022*	1.04

CI, confidence interval; VIF, Variance Inflation Factor; BS, Blood sugar.

*means *p-* value <0.05

### Correlations between amplitudes of flicker ERG and other health checkup factors

The results of univariable linear mixed effects model regression analyses with a random intercept for the amplitudes of the flicker ERGs and the 37 items of the health checkup are shown in Table 4. The items that were significantly correlated with the amplitudes of the flicker ERGs (*p-* values <0.05 after Holm-Bonferroni correction) were the age, the platelet (Plt) counts, and the creatinine (Cre) levels.

**Table 4:** Univariable linear mixed effects model regression analysis between amplitudes and explanatory variables.

	regression coefficient (β)	95% CI [0.025, 0.975]	*p-* value	*p-* value corrected using the Holm-Bonferroni method
BCVA (logMAR units)	-2.1	-3.3, -0.80	0.0015*	n.s.
IOP (mmHg)	3.0×10^−3^	-9.4×10^−2^, 0.10	0.95	n.s.
Axial length (mm)	-4.4×10^−2^	-0.25, 0.16	0.67	n.s.
Pupil Area (mm^2^)	3.3×10^−2^	-6.8×10^−2^, 0.13	0.52	n.s.
Sex-Male	-0.27	-0.75, 0.22	0.28	n.s.
Sex-Female	0.27	-0.22, 0.75	0.28	n.s.
Age (years)	-4.9×10^−2^	-7.4×10^−2^, -2.4×10^−2^	<0.001*	0.0047*
Height (cm)	1.6×10^−2^	-1.2×10^−2^, 4.5×10^−2^	0.26	n.s.
BMI (kg/m^2^)	-2.4×10^−2^	-9.6×10^−2^, 4.6×10^−2^	0.50	n.s.
Systolic BP (mmHg)	-3.8×10^−3^	-1.5×10^−2^, 7.8×10^−3^	0.52	n.s.
Diastolic BP (mmHg)	3.5×10^−3^	-1.4×10^−2^, 2.1×10^−2^	0.70	n.s.
average BP (mmHg)	-4.4×10^−4^	-1.6×10^−2^, 1.5×10^−2^	0.96	n.s.
Brinkman index (cigarettes・years/​day)	3.9×10^−5^	-5.6×10^−4^, 6.3×10^−4^	0.90	n.s.
IMT (mm)	-0.16	-0.59, 0.26	0.45	n.s.
WBC (10^3^/μL)	6.7×10^−2^	-9.5×10^−2^, 0.23	0.42	n.s.
RBC (10^4^/μL)	5.1×10^−3^	-8.8×10^−4^, 1.1×10^−2^	0.091	n.s.
Hb (g/dL)	0.18	-1.1×10^−2^, 0.37	0.065	n.s.
Ht (%)	5.9×10^−2^	-5.8×10^−3^, 0.12	0.075	n.s.
MCV (fL)	1.7×10^−3^	-4.8×10^−2^, 5.1×10^−2^	0.95	n.s.
Plt (10^4^/μL)	8.2×10^−2^	3.4×10^−2^, 0.13	<0.001*	0.031*
HbA1c (%)	-0.18	-0.66, 0.29	0.45	n.s.
BS (mg/dL)	1.3×10^−2^	-4.0×10^−3^, 3.1×10^−2^	0.13	n.s.
Protein (g/dL)	-0.43	-1.1, 0.19	0.17	n.s.
Alb (g/dL)	0.37	-0.62, 1.4	0.46	n.s.
ALP (U/L)	2.4×10^−3^	-1.4×10^−3^, 6.2×10^−3^	0.22	n.s.
GOT (U/L)	-2.9×10^−2^	-5.8×10^−2^, 2.5×10^−4^	0.053	n.s.
GPT (U/L)	-2.1×10^−3^	-1.8×10^−2^, 1.3×10^−2^	0.79	n.s.
γGTP (U/L)	6.7×10^−3^	1.9×10^−2^, 1.1×10^−2^	0.0058*	n.s.
Total cholesterol (mg/dL)	1.1×10^−2^	3.7×10^−3^,1.7×10^−2^	0.0026*	n.s.
TG (mg/dL)	-4.9×10^−4^	-4.4×10^−3^, 3.5×10^−3^	0.81	n.s.
HDL (mg/dL)	2.5×10^−2^	8.2×10^−3^, 4.1×10^−2^	0.0034*	n.s.
LDL (mg/dL)	4.5×10^−3^	-3.2×10^−3^, 1.2×10^−2^	0.26	n.s.
BUN (mg/dL)	-9.1×10^−2^	-0.15, -2.8×10^−2^	0.0047*	n.s.
Cre (mg/dL)	-2.6	-4.1, -1.2	<0.001*	0.011*
Uric acid (mg/dL)	-0.17	-0.35, 7.3×10^−3^	0.061	n.s.
Ca (mg/dL)	-0.11	-0.97, 0.75	0.80	n.s.
CRP (mg/dL)	-1.4	-2.5, -0.34	0.010*	n.s.

CI, confidence interval; BCVA, best-corrected visual acuity; IOP, Intraocular pressure; BMI, Body mass index; BP, Blood pressure; IMT, Intima Media Thickness; WBC, white blood cell; RBC, red blood cell; Hb, hemoglobin; Ht, hematocrit; MCV, mean corpuscular volume; Plt, Platelet; HbA1c, Hemoglobin A1c; BS, Blood sugar; Alb, albumin; ALP, alkaline phosphatase; GOT, glutamic oxaloacetic transaminase; GPT, glutamic pyruvic transaminase; γGTP, γ- glutamyl transpeptidase; TG, triglyceride; HDL, high density lipoprotein cholesterol; LDL, low density lipoprotein cholesterol; BUN, Blood urea nitrogen; Cre, Creatinine; Ca, calcium; CRP, C-reactive protein; n.s., not significant.

* means *p*- value <0.05

Adjusted P values for factors that were not significant in univariable models are not shown because they are also not significant after Holm-Bonferroni adjustment.

Backward stepwise model selection using Akaike information criterion (AIC) showed that the linear mixed effects regression models with explanatory variables of the age, the platelet count, and the creatinine levels best explain the variations of the amplitudes of the flicker ERGs; the age (*p* = 0.0084), platelet (*p* = 0.025), and creatinine (*p* = 0.011) were significantly correlated with the amplitudes of the fundamental components of the RETeval flicker ERGs (Table 5).

**Table 5:** Multivariable linear mixed effects model regression analysis between amplitudes and explanatory variables.

	Standardized partial regression coefficient (β)	Partial regression coefficient (B)	95% CI [0.025, 0.975]	*p*- value	VIF
Age (years)	-9.2×10^−2^	-3.5×10^−2^	-6.1×10^−2^, -9.0×10^−3^	0.0084*	1.10
Plt (10^4^/μL)	9.9×10^−2^	5.7×10^−2^	7.1×10^−3^, 0.11	0.025*	1.07
Cre (mg/dL)	-0.12	-1.9	-3.4, -0.45	0.011*	1.07

CI, confidence interval; VIF, Variance Inflation Factor; Plt, Platelet; Cre, Creatinine.

*means *p*-value<0.05

## Discussion

To the best of our knowledge, this is the first study that determined the significance of the correlations between an objective visual function recorded by RETeval flicker ERGs and blood tests or anthropometric findings for a large number of individuals undergoing a health checkup screening. The results of the univariable linear mixed effects model regression analysis showed that the BCVA, axial length, the age, blood sugar level, and BUN were significantly associated with the implicit times of the RETeval flicker ERGs. In addition, multivariable linear mixed effects model regression analysis showed that the age, the axial length, and the blood sugar levels were significantly correlated with the implicit times of the fundamental components of the RETeval flicker ERGs. Our analyses also showed that the age, platelet counts, and creatinine levels were significantly correlated with the amplitudes of the RETeval flicker ERGs. Because other studies have reported that the smoking habits (Brinkman index), BMI, and blood pressure were significantly correlated with the ocular findings [19–24], we examined whether these items were also significantly correlated with the different components of the flicker ERGs recorded with the RETeval system. Our analyses showed that none of these factors was significantly correlated with the components of the flicker ERGs recorded with the RETeval system.

Our results showed that the mean implicit time of the flicker ERGs was 32.0 ± 1.5 ms. This mean implicit times was slightly shorter than the 33.3 ± 1.3 ms or 33.2 ms reported in earlier studies [7, 12]. It is not clear whether this is a statistically significant difference, and the exact cause for this difference was not determined. However, a shorter axial length and smaller pupil size might be related to this difference. This is because the earlier study suggested that these factors tended to cause shorter implicit times of the flicker ERGs recorded with a RETeval flicker ERG system [12].

The mean amplitude of the flicker ERGs was about 70% of that of earlier studies recorded under the same stimulus conditions. This lower mean amplitude may be due to the difference in the age of the subjects. In the earlier study, the subjects were between 20- and 29-years-of-age [12], while our data were obtained from those who were ≥40-years-of-age. In support of this, Birch et al. reported that the amplitudes of the conventional flicker ERGs were smaller and the implicit times were prolonged in subjects who were about 70-years-of-age compared to those of around 20-years-of-age [25]. Our results also suggested that the amplitudes and implicit times were related to the age of the participants. These data may reflect the decline of the retinal function by the aging process.

The significant correlation between the axial length and the implicit times of the RETeval flicker ERGs has also been reported earlier [12]. The exact cause for the longer implicit times in eyes with longer axial lengths has not been determined, but Kato et al. suggested three possible factors [12]; a decrease in the retinal illuminance in eyes with longer axial lengths, an increase in the distance between the electrical signals in the retina and the electrodes, and a change in the retinal function caused by the stretching and thinning of the retina associated with the axial length elongations [26–31].

**Fig 2:**
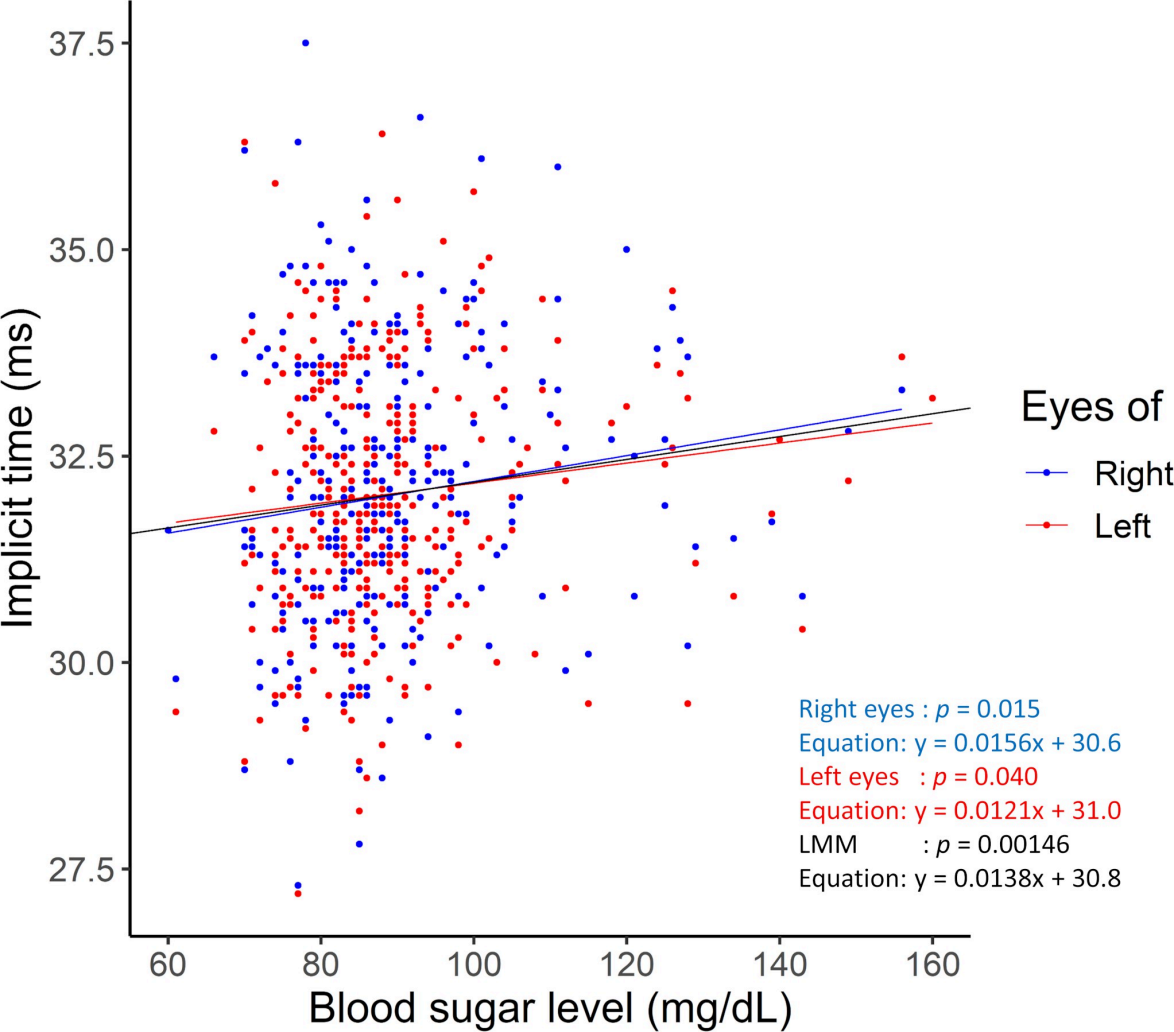
Relationship between blood sugar level and implicit times of the fundamental components of the RETeval flicker ERGs. The blood sugar levels are plotted against the implicit times of the fundamental component of the RETeval flicker ERGs. In the univariable linear regression analysis between the blood sugar level and implicit times of the fundamental components of the RETeval flicker ERGs, the *p* = 0.015 in the right eyes (blue plot and approximate line: y = 0.0156x + 30.6), *p* = 0.040 in the left eyes (red plot and approximate line: y = 0.0121x + 31.0), and *p* = 0.00146 in the linear mixed effects model (black approximate line: y = 0.0138x + 30.8). LMM, linear mixed effects model.

Earlier studies have shown that the prolongation of the implicit times of the flicker ERGs recorded by RETeval was a useful marker of diabetes diabetic retinopathy [7, 32, 33]. In our study, we analyzed only 23 participants whose HbA1c was ≥6.5% which is a diagnostic criterion for diabetes [34]. The correlation between HbA1c and the implicit time of the RETeval flicker ERG was not significant (*p* = 0.48; adjusted by Holm-Bonferroni method). This finding of non-significance by the Holm-Bonferroni correlation may be because of the smaller sample size of the participants with diabetes. On the other hand, the significant correlation between the blood sugar level and the implicit times in the univariable linear mixed effects model regression analysis (the blood sugar levels are plotted against the implicit times of the fundamental component of RETeval flicker ERGs in Fig 2) suggested that a transient elevation of blood sugar level may lead to a prolongation in the implicit times even in non-diabetic individuals.

Although the backward stepwise model selection did not detect the significant correlations between the implicit times and the BCVA and BUN, this may be related to the longer implicit times of the flicker ERGs. Basically, we studied participants with normal OCT and fundus findings, and therefore the relationship between the BCVA and implicit times of the flicker ERGs suggested two possibilities; one is a subclinical damage of the retina and another is the presence of cataracts which cause a reduction of the BCVA and a delay of the implicit time due to a reduction of the flash intensity as reported [35].

We did not expect the BUN level would be significantly correlated with the implicit times and that the Cre level would be significantly correlated with the amplitudes of the flicker ERGs recorded with the RETeval system in the regression analysis. These factors were not highly correlated with age and other explanatory variables considering the VIF, therefore we believe that they are independent factors that were significantly correlated with the flicker ERGs. The BUN level and Cre level are related to renal function, and they have been reported to be associated with slightly reduced 30-Hz flicker ERGs after long-term hemodialysis [36]. A significant correlation between chronic kidney disease and age-related macular degeneration has also been reported [37–39], and some reports suggested that drusen, the precursor of age-related macular degeneration, may have the same composition as glomerular deposits [40–42]. A decreased renal function associated with potential and pre-clinical glomerular dysfunction may be correlated with the decreased ERG components associated with deposition and its incomplete discharge between the retinal pigment epithelial cells and Bruch’s membrane. However, our study did not include participants with severe renal failure (estimated glomerular filtration rate: eGFR <29). Thus, the reason for the significant correlations between the implicit times and the BUN level and the amplitudes and Cre level were not definitively determined.

It was also unexpected that the Plt count would be significantly correlated with the amplitudes of the flicker ERGs recorded with the RETeval system in the regression analysis. This study was designed to find new significant correlations between the RETeval flicker ERGs and the hematologic tests and anthropometric data of normal individuals, and such new correlations were found. However, our findings showed that the correlations between the red blood cells counts and implicit times and amplitudes of the flicker ERGs were not significant, and there was also no significant correlation between the platelet counts and the implicit times of the flicker ERGs. We suggest this finding was may not due to a reduction of retinal function associated with the oxygen supply or demand of the retina but due to other factors, such as changes of the skin conductivity due to platelet-related skin quality or conditions that affect the fundamental components of the flicker ERGs. The main function of platelets is to contribute to coagulation and hemostasis, but it has been pointed out that they also secrete growth factors such as the platelet-derived growth factor, vascular endothelial growth factor, epidermal growth factor, and transforming growth factor-β (TGF-β) [43–46]. It has also been reported that TGF-β may contribute to epidermal and dermal thickening and cell turnover in skin wound healing [47–49]. It is also possible that increased platelet counts may have led to better skin conditions and higher skin conductivity. However, data on the skin conditions of the participants were not collected in this study. Future studies are needed to confirm the significant found detected in this study.

The variance of the amplitudes of the flicker ERGs was larger than that of the implicit times; the relative standard deviation (RSD) was 1.5/32 = 0.05 for the implicit times and 3.2/9.73 = 0.33 for the amplitudes. The reproducibility should be checked by future studies.

There are several limitations in this study. First, the participants were all ≥40-years-of-age who lived in a relatively rural area where many people had jobs in agriculture and fishing. The differences of the life style from people in an urban environment, in which myopia is prevalent due to display work or near work [50], would be expected to affect the results. Second, we analyzed 37 explanatory variables. To guard against false positive findings, the Holm-Bonferroni method was used to reduce the Type 1 error rate.

In conclusion, our data analyses identified age and several ophthalmologic and hematologic factors that were significantly associated with the retinal electrophysiological activity. The influences of the axial length, the age, blood sugar level, and possibly the BCVA and BUN on the implicit times of RETeval flicker ERGs should be considered when evaluating the retinal function in the health screenings. In addition, the amplitudes might be affected by the age, Plt count, and Cre levels, although anthropometric and lifestyle habits may not be significantly correlated with the retinal function.

## Supporting information

S1 Table Reasons of known ocular diseases or abnormal findings for the exclusion. (PDF)

S2 Table The data items studied of health checkup. (PDF)

S3 Table Results of hematologic tests. (PDF)

S1 File Original data used in this article. (XLSX)
