# Supplementary material for: Assessment of factors affecting flicker ERGs recorded with RETeval from data obtained from health checkup screening
Source: PLoS One. 2023 Apr 24;18(4):e0284686. doi: 10.1371/journal.pone.0284686 (PMC10124871; doi:10.1371/journal.pone.0284686)
Supplement: S1 Table — (PDF) [file pone.0284686.s001.pdf]

S1 Table. Reasons of known ocular diseases or abnormal findings for the exclusion

	Right eyes	Left eyes
glaucoma or glaucoma suspect	46	55
drusen	20	17
epiretinal membrane	11	10
age-related macular degeneration	11	8
unreadable fundus color	6	12
fundus hemorrhage	4	6
myopic conus	3	6
retinal edema	3	2
retinal degeneration	2	4
diabetic retinopathy	2	2
retinoschisis	2	2
after retinal photocoagulation	2	1
macular hole	1	0
vitreomacular traction syndrome	1	0

The left panel shows the reasons of known ocular diseases or abnormal findings for the exclusion, middle panel shows the number of the reasons of the left panel in the right eyes, and the right panel shows the number of the reasons of the left panel in the left eyes.
