# Supplementary material for: Assessment of factors affecting flicker ERGs recorded with RETeval from data obtained from health checkup screening
Source: PLoS One. 2023 Apr 24;18(4):e0284686. doi: 10.1371/journal.pone.0284686 (PMC10124871; doi:10.1371/journal.pone.0284686)
Supplement: S2 Table — (PDF) [file pone.0284686.s002.pdf]

S2 Table. The data items studied of health checkup

BCVA	logMAR	-
IOP	mmHg	-
Axial length	mm	-
Radius of the pupillary diameter	mm	-
Amplitude of the flicker ERGs	μV	-
Implicit time of the flicker ERGs	msec	-
Sex	Male / Female	-
Age	years	-
Height	cm	-
Mass	kg	-
Systolic blood pressure	mmHg	-
Diastolic blood pressure	mmHg	-
Brinkman index	cigarettes・years/day	-
IMT	mm	≦1.0
WBC	10^3/mL	3.3 – 8.6
RBC	10^4/mL	Male: 435 – 555 / Female: 386 – 492
Hb	g/dL	Male: 13.7 – 16.8 / Female: 11.6 – 14.8
Ht	%	Male: 40.7 – 50.1 / Female: 35.1 – 44.4
MCV	fL	83.6 – 98.2
Plt	10^4/mL	15.8 – 34.8
HbA1c	%	4.9 – 6.0
BS	mg/dL	73 – 109
Protein	g/dL	6.6 – 8.1
Alb	g/dL	4.1– 5.1
ALP	U/L	106 – 322
GOT	U/L	13 – 30
GPT	U/L	Male: 10 – 42 / Female: 7 – 23
γGTP	U/L	Male: 13 – 64 / Female: 9 – 32
Total cholesterol	mg/dL	142 – 248
TG	mg/dL	Male: 40 – 234 / Female: 30 – 117
HDL	mg/dL	Male: 38 – 90
LDL	mg/dL	65 – 163
BUN	mg/dL	8 – 20
Cre	mg/dL	Male: 0.65 – 1.07 / Female: 0.46 – 0.79
Uric acid	mg/dL	Male: 3.7 – 7.8 / Female: 2.6 – 5.5
Ca	mg/dL	8.8 – 10.1
CRP	mg/L	0.00 – 0.14

The left panel shows the data item studied of health checkup, middle panel shows units or classifications of the items, and the right panel shows the reference intervals published by Japanese Society of Laboratory Medicine (JSLM, 2018).

BCVA, best-corrected visual acuity; IOP, Intraocular pressure; IMT, Intima Media Thickness; WBC, white blood cell; RBC, red blood cell; Hb, hemoglobin; Ht, hematocrit; MCV, mean corpuscular volume; Plt, Platelet; HbA1c, Hemoglobin A1c; BS, blood sugar; Alb, albumin; ALP, alkaline phosphatase; GOT, glutamic oxaloacetic transaminase; GPT, glutamic pyruvic transaminase; γGTP, γ- glutamyl transpeptidase; TG, triglyceride; HDL, high density lipoprotein cholesterol; LDL, low density lipoprotein cholesterol; BUN, Blood urea nitrogen; Cre, Creatinine; Ca, calcium; CRP, C-reactive protein.
