# Supplementary material for: Assessment of factors affecting flicker ERGs recorded with RETeval from data obtained from health checkup screening
Source: PLoS One. 2023 Apr 24;18(4):e0284686. doi: 10.1371/journal.pone.0284686 (PMC10124871; doi:10.1371/journal.pone.0284686)
Supplement: S3 Table — (PDF) [file pone.0284686.s003.pdf]

S3 Table. Results of hematologic tests

WBC (10^3/μL)	5.91 ± 1.49 [2.9-13.2]
RBC (10^4/μL)	447 ± 40 [310-586]
Hb (g/dL)	13.5 ± 1.2 [7.8-17.7]
Ht (%)	41.3 ± 3.7 [26.2-53.1]
MCV (fL)	92.5 ± 4.9 [64.3-111]
Plt (10^4/μL)	21.9 ± 4.9 [9.3-41.7]
HbA1c (%)	5.7 ± 0.51 [4.0-8.9]
BS (mg/dL)	88 ± 14 [60-160]
Protein (g/dL)	7.3 ± 0.39 [6.3-8.5]
Alb (g/dL)	4.3 ± 0.24 [3.5-5.0]
ALP (U/L)	214 ± 63 [78-543]
GOT (U/L)	23.3 ± 8.2 [12-87]
GPT (U/L)	23.6 ± 15.5 [7-162]
γGTP (U/L)	35.8 ± 50.4 [7-474]
Total cholesterol (mg/dL)	214 ± 35 [115-363]
TG (mg/dL)	105 ± 61 [30-475]
HDL (mg/dL)	59.5 ± 14.5 [32-125]
LDL (mg/dL)	126 ± 31.0 [50-263]
BUN (mg/dL)	14.7 ± 3.8 [5.4-29.2]
Cre (mg/dL)	0.75 ± 0.17 [0.43-1.55]
Uric acid (mg/dL)	5.38 ± 1.33 [2.2-9.7]
Ca (mg/dL)	9.38 ± 0.28 [8.6-10.1]
CRP (mg/dL)	0.09 ± 0.22 [0.002-2.626]

WBC, white blood cell; RBC, red blood cell; Hb, hemoglobin; Ht, hematocrit; MCV, mean corpuscular volume; Plt, Platelet; HbA1c, Hemoglobin A1c; BS, Blood sugar; Alb, albumin; ALP, alkaline phosphatase; GOT, glutamic oxaloacetic transaminase; GPT, glutamic pyruvic transaminase; γGTP, γ- glutamyl transpeptidase; Total Chol, Total cholesterol; TG, triglyceride; HDL, high density lipoprotein cholesterol; LDL, low density lipoprotein cholesterol; BUN, Blood urea nitrogen; Cre, Creatinine; Ca, calcium; CRP, C-reactive protein.

Data are mean ± standard deviation [range].
